# Efficacy of blood copeptin level for the prediction of mortality of adult patients with sepsis: a meta-analysis

**DOI:** 10.3389/fmed.2025.1686137

**Published:** 2026-02-06

**Authors:** Jiayang Huang, Tingting Niu, Huijie Shi

**Affiliations:** 1 Department of Pharmacy, Shenzhen People’s Hospital, Shenzhen, China; 2 The Second Clinical Medical College, Jinan University, Shenzhen, China; 3 The First Affiliated Hospital, Southern University of Science and Technology, Shenzhen, China

**Keywords:** copeptin, blood, sepsis, mortality, prediction

## Abstract

**Background:**

Copeptin, the C-terminal fragment of provasopressin, has emerged as a potential prognostic biomarker in sepsis. However, its predictive accuracy for mortality in adult patients with sepsis remains uncertain. We conducted a systematic review and meta-analysis to evaluate the diagnostic performance of elevated blood copeptin levels for mortality prediction in this population.

**Methods:**

We systematically searched PubMed, Embase, Web of Science, Wanfang Data, and CNKI from inception to 22 May 2025, for observational studies assessing copeptin levels at admission or within 48 h in adults with sepsis. Pooled sensitivity, specificity, likelihood ratios, diagnostic odds ratio (DOR), and area under the summary receiver operating characteristic curve (AUC) were calculated using a random-effects model. Study quality was assessed using QUADAS-2.

**Results:**

Ten prospective studies involving 1,637 patients were included. Pooled sensitivity and specificity of elevated copeptin for predicting mortality were 0.77 (95% CI: 0.70–0.83; I^2^ = 52%) and 0.76 (95% CI: 0.67–0.83; I^2^ = 86%), respectively. The pooled positive and negative likelihood ratios were 3.16 (95% CI: 2.33–4.29) and 0.30 (95% CI: 0.23–0.40), with a DOR of 10.40 (95% CI: 6.62–16.33). The summary AUC was 0.83 (95% CI: 0.79–0.86), indicating good overall prognostic accuracy. Subgroup analysis according to the cutoffs of copeptin did not significantly affect the results. No significant publication bias was detected (*p* = 0.58).

**Conclusion:**

Elevated blood copeptin levels within 48 h of sepsis diagnosis show good prognostic accuracy for short-term mortality in adult patients with sepsis. These findings support the potential clinical utility of copeptin as a risk stratification tool in sepsis management.

**Systematic review registration:**

https://www.crd.york.ac.uk/prospero/, identifier CRD42024587540.

## Introduction

Sepsis is a life-threatening organ dysfunction caused by a dysregulated host response to infection and remains a leading cause of morbidity and mortality worldwide (1, 2). It affects millions of people annually, with a mortality rate ranging from 15% to over 40% depending on disease severity, comorbidities, and healthcare resources (3). Despite advances in antimicrobial therapy, hemodynamic support, and critical care management, the prognosis of sepsis remains poor, particularly among patients with septic shock or multiple organ failure (4). Current prognostic assessment in sepsis often relies on clinical severity scores such as the Acute Physiology and Chronic Health Evaluation II (APACHE II) and Sequential Organ Failure Assessment (SOFA) (5), alongside conventional biomarkers like procalcitonin and C-reactive protein (6). However, these parameters have limitations in sensitivity, specificity, and timeliness, highlighting the need to identify novel biomarkers that can reliably predict patient outcomes and facilitate early risk stratification (7, 8).

Copeptin, the C-terminal portion of provasopressin, is a 39-amino acid glycopeptide released in equimolar amounts with arginine vasopressin (AVP) from the posterior pituitary (9, 10). Unlike AVP, copeptin is highly stable in plasma and can be easily measured, serving as a surrogate marker for AVP release (11). AVP plays a key role in vasoconstriction, water homeostasis, and stress response, and elevated copeptin levels reflect activation of the hypothalamic–pituitary–adrenal axis and the body’s stress adaptation system (12, 13). In sepsis, excessive AVP and copeptin release may be driven by severe infection, systemic inflammation, and hemodynamic instability, and high levels have been associated with circulatory failure, organ dysfunction, and death (14). Several observational studies have investigated the prognostic value of copeptin in sepsis, but results have varied, with differences in patient populations, diagnostic criteria, timing of measurement, and cutoff thresholds (15–24). Given these inconsistencies and the absence of a comprehensive quantitative synthesis of the evidence, we performed a systematic review and meta-analysis of observational studies to evaluate the prognostic performance of elevated blood copeptin levels for predicting mortality in adult patients with sepsis.

## Materials and methods

This systematic review and meta-analysis was conducted in accordance with the (preferred reporting items for systematic reviews and meta-analyses) PRISMA guidelines (25, 26) and followed methodological recommendations provided in the Cochrane Handbook (25) to ensure rigor in study design, data synthesis, and reporting. The protocol of the meta-analysis has been registered in PROSPERO with the ID: CRD42024587540.

### Database search

To identify eligible studies, we conducted a systematic search of PubMed, Embase, Web of Science, Wanfang Data and CNKI (China National Knowledge Infrastructure) using a combination of terms related to the biomarker (“copeptin” OR “C-terminal provasopressin”), and disease condition (“sepsis” OR “septic” OR “septicemia”). No outcome-related search terms were included, as we sought to broaden the search strategy to avoid missing potentially relevant studies. The search was limited to human studies published as full-text articles in English or Chinese, covering the period from database inception to 22 May 2025. Additionally, we manually screened the reference lists of relevant publications to identify further eligible studies. The complete search strategies for each database are provided in Supplementary File 1.

### Study selection criteria

Studies were selected if they met the following criteria:

Population (P): Adults (≥ 18 years) with a diagnosis of sepsis, severe sepsis, or septic shock according to recognized criteria.

Index test (I): Measurement of blood copeptin levels at admission or within the first 48 h of sepsis diagnosis. Any assay method accepted.

Comparator (C): Reference standard: confirmed mortality outcome at a defined time point (e.g., in-hospital mortality, 28-day mortality, mortality during intensive care unit [ICU] or hospitalization). Studies without a comparator group were still eligible if they report diagnostic accuracy parameters.

Outcomes (O): Sufficient data to construct a 2 × 2 table (true positives, false positives, false negatives, true negatives) for mortality prediction at a given cutoff of copeptin, or reported sensitivity, specificity, and/or area under the receiver operating characteristic (ROC) curve (AUC) with 95% confidence interval [CI]. If multiple thresholds are reported, extract the one defined as “optimal” by the study (e.g., Youden index).

Study design (S): Observational studies.

Studies were excluded if either of the criteria was met: (1) Studies in pediatric or neonatal populations; (2) Studies without mortality as an outcome; (3) Studies not reporting copeptin measurements at baseline (e.g., post-treatment); (4) Reviews, editorials, conference abstracts without full data, animal studies, or pre-clinical research; (5) Duplicate data from the same cohort (include the most complete/updated dataset); or (6) Studies without sufficient data to calculate diagnostic accuracy measures. If two studies included potentially overlapping patient populations, the one with the largest sample size was included in the meta-analysis.

### Data collection and quality assessment

Two independent reviewers screened the literature, extracted data, and assessed study quality using predefined criteria. Discrepancies were resolved through discussion until consensus was reached. Extracted data included study characteristics (first author, publication year, country, and design), patient diagnosis and diagnostic criteria for sepsis, patient characteristics (sample size, mean age, and sex distribution), timing and methods of blood copeptin measurement, copeptin cutoff values, follow-up durations, methods of mortality verification, and true positive (TP), false positive (FP), false negative (FN), and true negative (TN) counts for mortality prediction in sepsis. Study quality was assessed using the Quality Assessment Tool for Diagnostic Accuracy Studies (QUADAS-2) tool (27), with each study rated as having low, high, or unclear risk of bias across key domains based on risk sources and applicability.

### Statistical methods

Sensitivity, specificity, and positive and negative diagnostic likelihood ratios (DLRs) for the predictive value of elevated blood copeptin in assessing mortality risk among patients with sepsis were pooled from 2 × 2 tables, together with their corresponding 95% CI. The diagnostic odds ratio (DOR), indicating the odds of a correct diagnosis relative to a misdiagnosis (28), was also calculated to reflect overall test accuracy. Discriminative ability was evaluated by pooling the area under the receiver operating characteristic curve (AUC) across studies. Between-study heterogeneity was assessed using the Cochrane Q test (*p* < 0.10 considered significant) (25), and quantified with the I^2^ statistic, with thresholds of < 25%, 25–75%, and > 75% indicating low, moderate, and substantial heterogeneity, respectively (29). A random-effects model was used to account for potential heterogeneity between studies (25). In addition, to explore whether differences in predefined copeptin thresholds influenced diagnostic performance, we conducted a subgroup analysis stratified by cutoff values (≤ 50 pmol/L vs. > 50 pmol/L), based on the medians of copeptin cutoffs used in the included studies. Pooled sensitivity, specificity, and AUC were recalculated within each subgroup. Publication bias was examined using Deeks’ funnel plot and asymmetry test (30). All statistical analyses were conducted using STATA software (Version 17.0; Stata Corporation, College Station, TX, USA), with *p* < 0.05 regarded as statistically significant.

## Results

### Results of literature search

**FIGURE 1:**
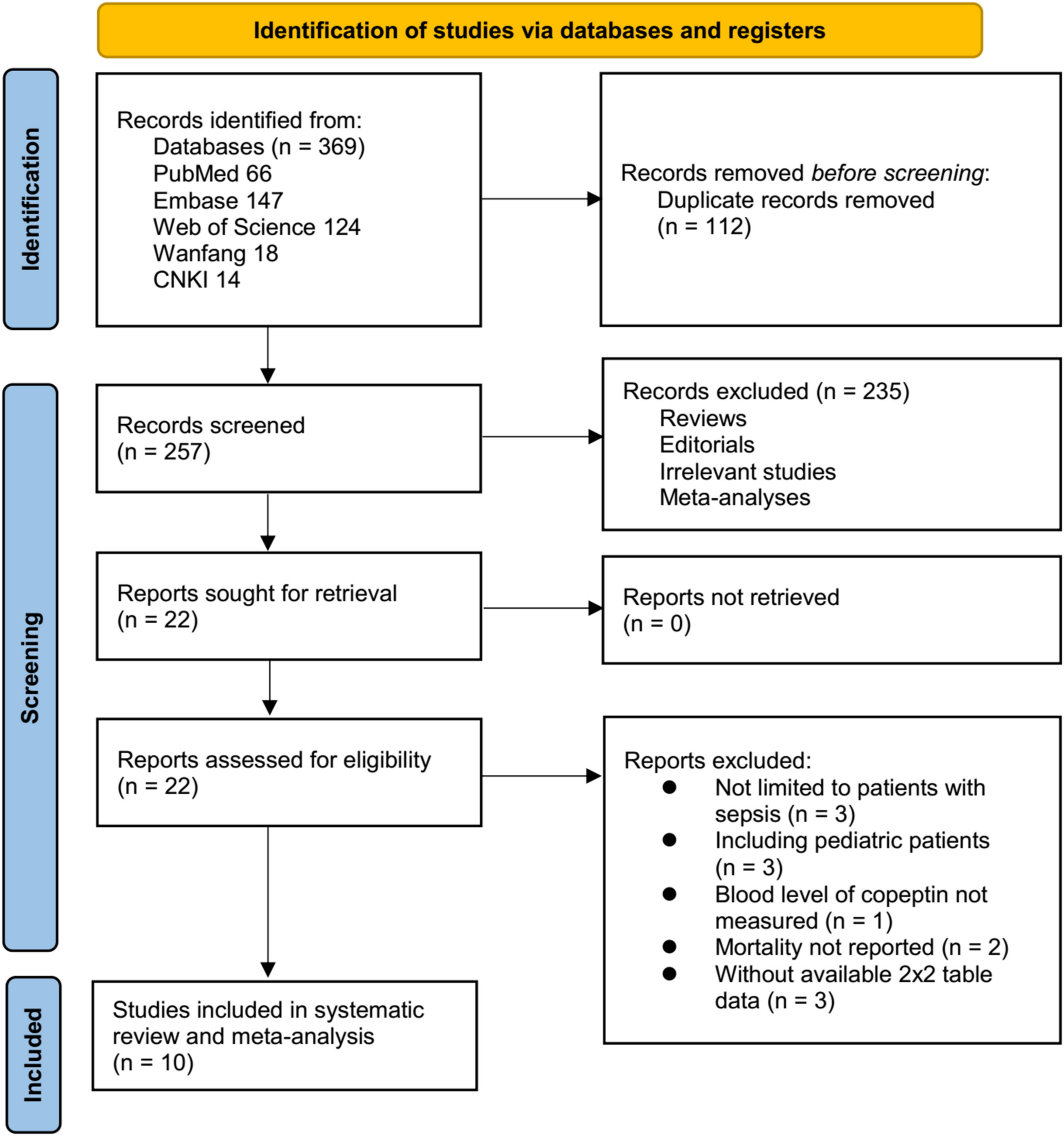
Flowchart illustrating the process of study identification, screening, eligibility assessment, and inclusion.

The initial database search yielded 369 studies as depicted in Figure 1, of which 257 remained after 112 duplicates were removed. Upon analyzing the titles and abstracts, a further 235 studies were excluded due to lack of relevance to the meta-analysis objective, leaving 22 studies undergoing full-text review. After a thorough review of full texts, 12 out of the remaining studies were excluded for reasons detailed in Figure 1. Ultimately, ten studies (15–24) were included for the meta-analysis.

### Study characteristics and quality assessment

The main characteristics of the 10 studies included in this meta-analysis are presented in Table 1. These prospective studies were conducted in China, Belgium, Italy, Egypt, Switzerland, and Turkey, and were published between 2014 and 2023. All studies enrolled adult patients with sepsis, severe sepsis, or septic shock, diagnosed according to Sepsis-1.0 (15, 17–21), Sepsis-2.0 (16, 23), or Sepsis-3.0 criteria (22, 24), respectively. The number of patients with sepsis per study ranged from 39 to 645, with an overall sample size of 1,637 of the meta-analysis. The mean age of participants varied between 54.7 and 72.0 years, with the proportion of male participants ranging from 39.3% to 66.7%. Blood copeptin levels were measured at admission or within 48 h using enzyme-linked immunosorbent assay (ELISA) (15, 16, 18, 19, 21–23), immunoluminometric assay (ILMA) (17), or fluorescent immunoassay (FIA) (20, 24). Cutoff values for copeptin varied from 11 to 145 pmol/L. Follow-up durations ranged from 7 to 30 days, and mortality was validated using medical records and/or follow-up. No study provided data on long-term mortality beyond 30 days. Study quality was assessed using the QUADAS-2 tool, with results detailed in Table 2. All studies were judged as having low risk of bias in the domains of patient selection and reference standard. Two studies (16, 19) had unclear risk in the index test domain, while one study (17) had unclear risk in the flow and timing domain due to insufficient information. All remaining domains, including those related to applicability concerns, were rated as low risk for all studies.

**TABLE 1:** Characteristics of the included studies.

References	Country	Design	Diagnosis	Criteria for sepsis	No. of patients with sepsis	Mean age (years)	Men (%)	Timing of copeptin measurement	Methods for copeptin measurement	Cutoff value of copeptin (pmol/L)	Follow-up duration (days)	Methods for validation mortality	TP	FP	FN	TN
Zhang et al. (15)	China	P	Sepsis, severe sepsis, or septic shock	Sepsis-1.0	461	72	61	At admission	ELISA	86.3	28	Medical records/follow-up	57	38	31	335
Jiang et al. (16)	China	P	Sepsis	Sepsis-2.0	41	62.1	61	At admission	ELISA	30	28	Medical records/follow-up	20	5	6	10
Laribi et al. (17)	Belgium	P	Severe sepsis, septic shock	Sepsis-1.0	99	66	66.7	Within 24 h of admission	ILMA	104	28	Medical records/follow-up	22	25	10	42
Battista et al. (20)	Italy	P	Sepsis, severe sepsis, or septic shock	Sepsis-1.0	64	70.5	53.1	At admission	FIA	23.2	30	Medical records/follow-up	14	6	5	39
Assaad et al. (19)	Egypt	P	Severe sepsis, septic shock	Sepsis-1.0	40	54.7	55	Within 48 h of admission	ELISA	11	10	Medical records/follow-up	11	5	8	16
Ameen et al. (18)	Egypt	P	Severe sepsis, septic shock	Sepsis-1.0	39	61.2	53.8	Within 24 h of admission	ELISA	145	28	Medical records/follow-up	14	5	3	17
Sobhy et al. (21)	Egypt	P	Sepsis, severe sepsis, or septic shock	Sepsis-1.0	60	57	46.7	At admission	ELISA	58.1	7	Medical records/follow-up	28	12	1	19
Kloter et al. (22)	Switzerland	P	Sepsis	Sepsis-3.0	645	61	55.8	At admission	ELISA	50	30	Medical records/follow-up	38	152	7	457
Cai et al. (23)	China	P	Sepsis	Sepsis-2.0	128	56.9	49.2	At admission	ELISA	27.9	30	Medical records/follow-up	26	16	6	80
Cander et al. (24)	Turkey	P	Sepsis or septic shock	Sepsis-3.0	60	64.1	39.3	Within 24 h of admission	FIA	73	30	Medical records/follow-up	21	16	7	16

P, prospective; TP, true positive; FP, false positive; FN, false negative; TN, true negative; ELISA, enzyme-linked immunosorbent assay; ILMA, immunoluminometric assay; FIA, fluorescent immunoassay.

**TABLE 2:** Study quality evaluation via the QUADAS-2 Scale.

References	Risk of bias				Applicability concerns		
	Patient selection	Index test	Reference standard	Flow and timing	Patient selection	Index test	Reference standard
Zhang et al. (15)	Low risk	Low risk	Low risk	Low risk	Low risk	Low risk	Low risk
Jiang et al. (16)	Low risk	Unclear	Low risk	Low risk	Low risk	Low risk	Low risk
Laribi et al. (17)	Low risk	Low risk	Low risk	Unclear	Low risk	Low risk	Low risk
Battista et al. (20)	Low risk	Low risk	Low risk	Low risk	Low risk	Low risk	Low risk
Assaad et al. (19)	Low risk	Unclear	Low risk	Low risk	Low risk	Low risk	Low risk
Ameen et al. (18)	Low risk	Low risk	Low risk	Low risk	Low risk	Low risk	Low risk
Sobhy et al. (21)	Low risk	Low risk	Low risk	Low risk	Low risk	Low risk	Low risk
Kloter et al. (22)	Low risk	Low risk	Low risk	Low risk	Low risk	Low risk	Low risk
Cai et al. (23)	Low risk	Low risk	Low risk	Low risk	Low risk	Low risk	Low risk
Cander et al. (24)	Low risk	Low risk	Low risk	Low risk	Low risk	Low risk	Low risk

QUADAS-2, quality assessment of diagnostic accuracy studies 2.

### Performance of high blood copeptin level for prediction of mortality

**FIGURE 2:**
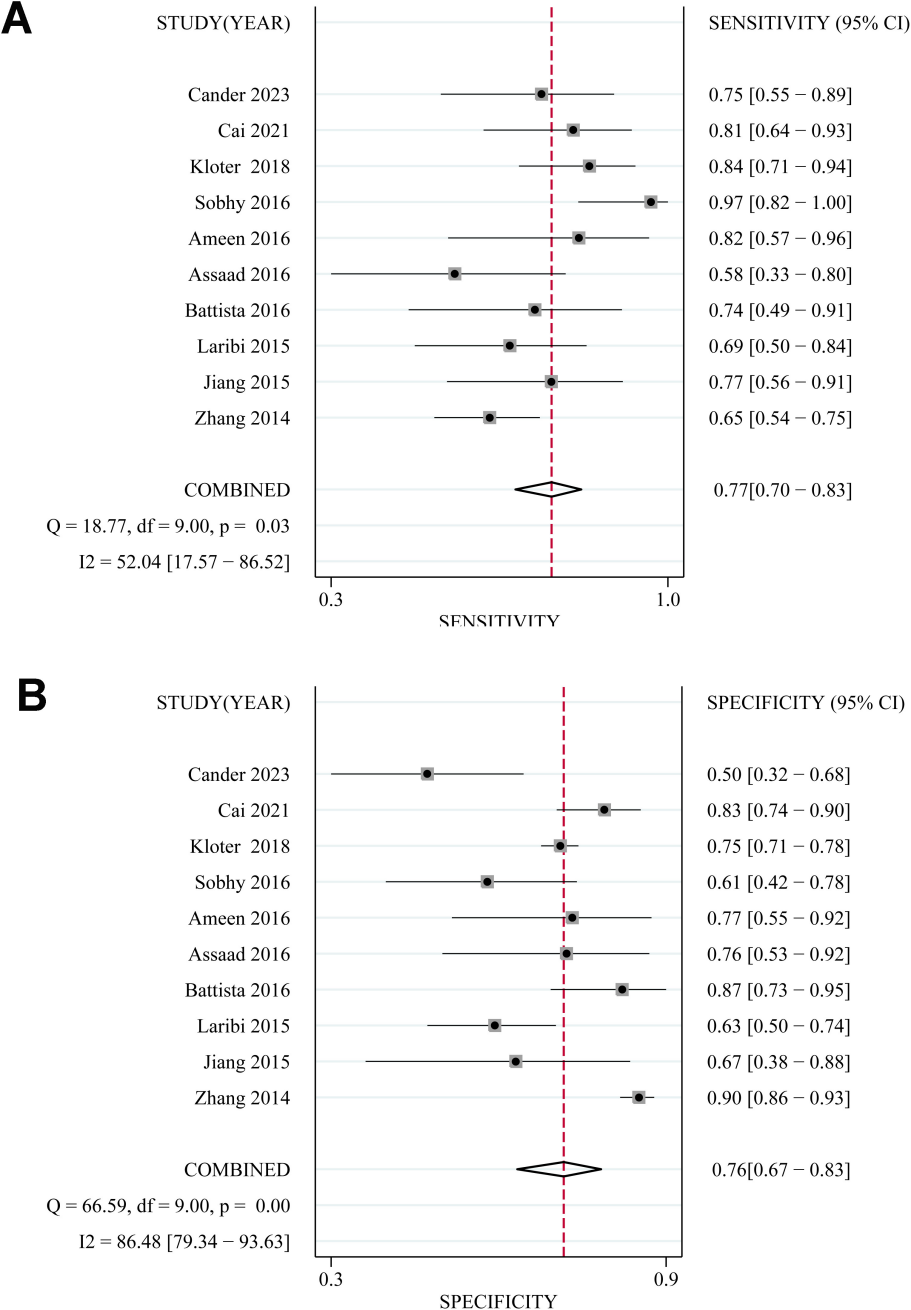
Forest plots showing the diagnostic performance of elevated blood copeptin levels for predicting mortality in adult patients with sepsis: **(A)** pooled sensitivity; and **(B)** pooled specificity.

**FIGURE 3:**
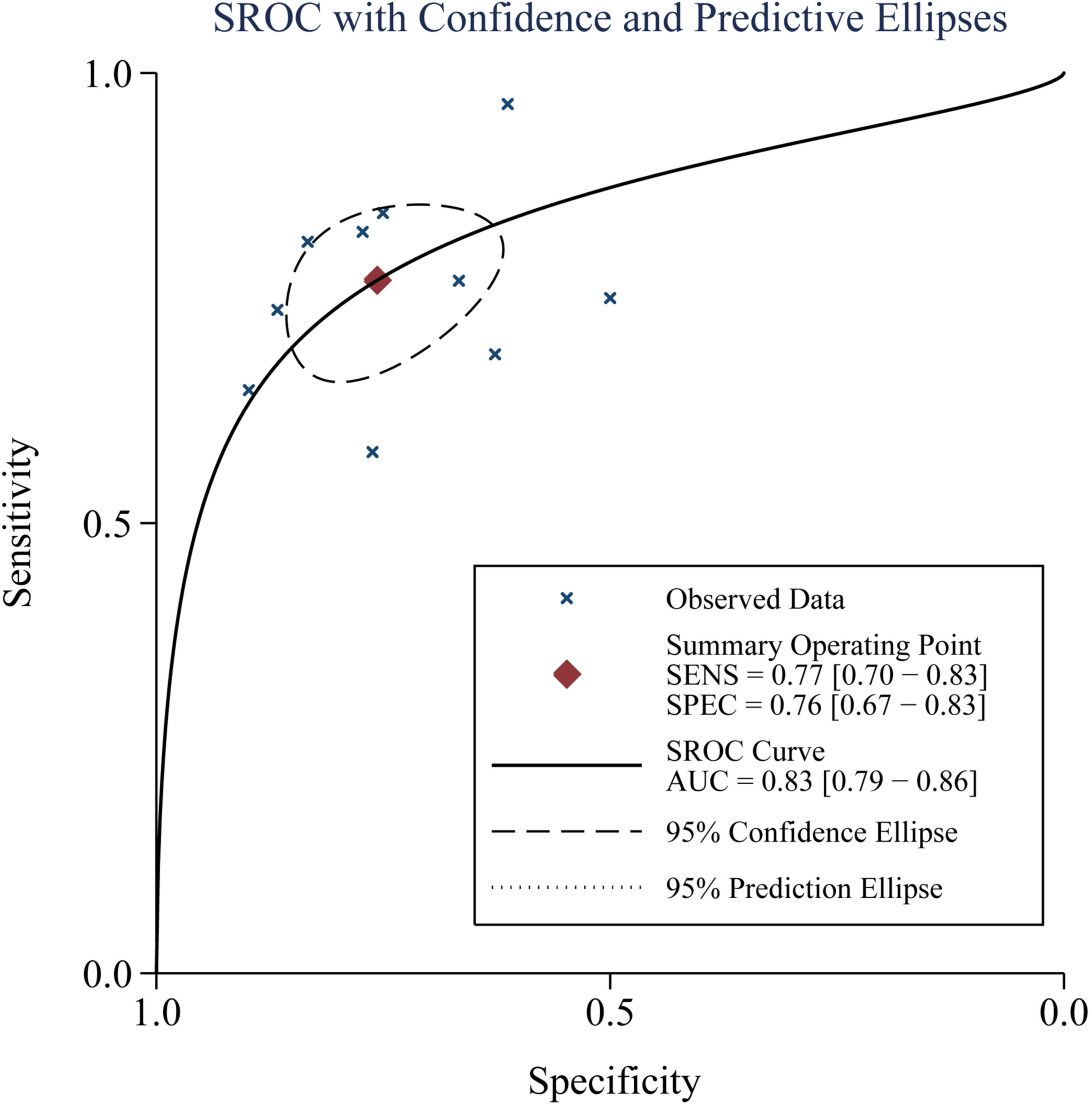
Summary receiver operating characteristic (SROC) curve showing the performance of elevated blood copeptin levels for predicting mortality in adult patients with sepsis.

Based on pooled data from 10 studies (15–24), a high blood copeptin level within 48 h after the diagnosis of sepsis demonstrated moderate diagnostic performance for predicting mortality risk within 30 days. The combined sensitivity and specificity were 0.77 (95% CI: 0.70–0.83; I^2^ = 52%, Figure 2A) and 0.76 (95% CI: 0.67–0.83; I^2^ = 86%, Figure 2B), respectively. The pooled positive and negative DLRs were 3.16 (95% CI: 2.33–4.29) and 0.30 (95% CI: 0.23–0.40), respectively, yielding a diagnostic odds ratio (DOR) of 10.40 (95% CI: 6.62–16.33). The area under the summary receiver operating characteristic curve (AUC) was 0.83 (95% CI: 0.79–0.86; Figure 3). Subgroup analysis based on copeptin thresholds is presented in Table 3. Studies using a cutoff value ≤ 50 pmol/L showed similar pooled sensitivity (0.76) and specificity (0.78) to those applying cutoffs > 50 pmol/L (sensitivity 0.75; specificity 0.71), and the between-group differences were not statistically significant. However, the specificity estimates varied more substantially among studies applying higher thresholds, suggesting that differences in cutoff selection may partially contribute to heterogeneity, particularly in specificity.

**TABLE 3:** Summarized predictive efficacy of blood copeptin for mortality of patients with sepsis according to the cutoff value of copeptin.

Cutoff of copeptin (pmol/L)	No. of study	Sensitivity			Specificity			AUC	
		Pooled value and 95% CI	I^2^	*p* for group difference	Pooled value and 95% CI	I^2^	*p* for group difference	Pooled value and 95% CI	*p* for group difference
≤ 50	5	0.76 (0.67–0.84)	27%		0.78 (0.72–0.83)	37%		0.73 (0.34–0.86)	
> 50	5	0.75 (0.62–0.84)	54%	0.22	0.71 (0.48–0.86)	93%	0.34	0.62 (0.26–0.82)	0.49

AUC, area under the summary receiver operating characteristic curve.

### Publication bias

**FIGURE 4:**
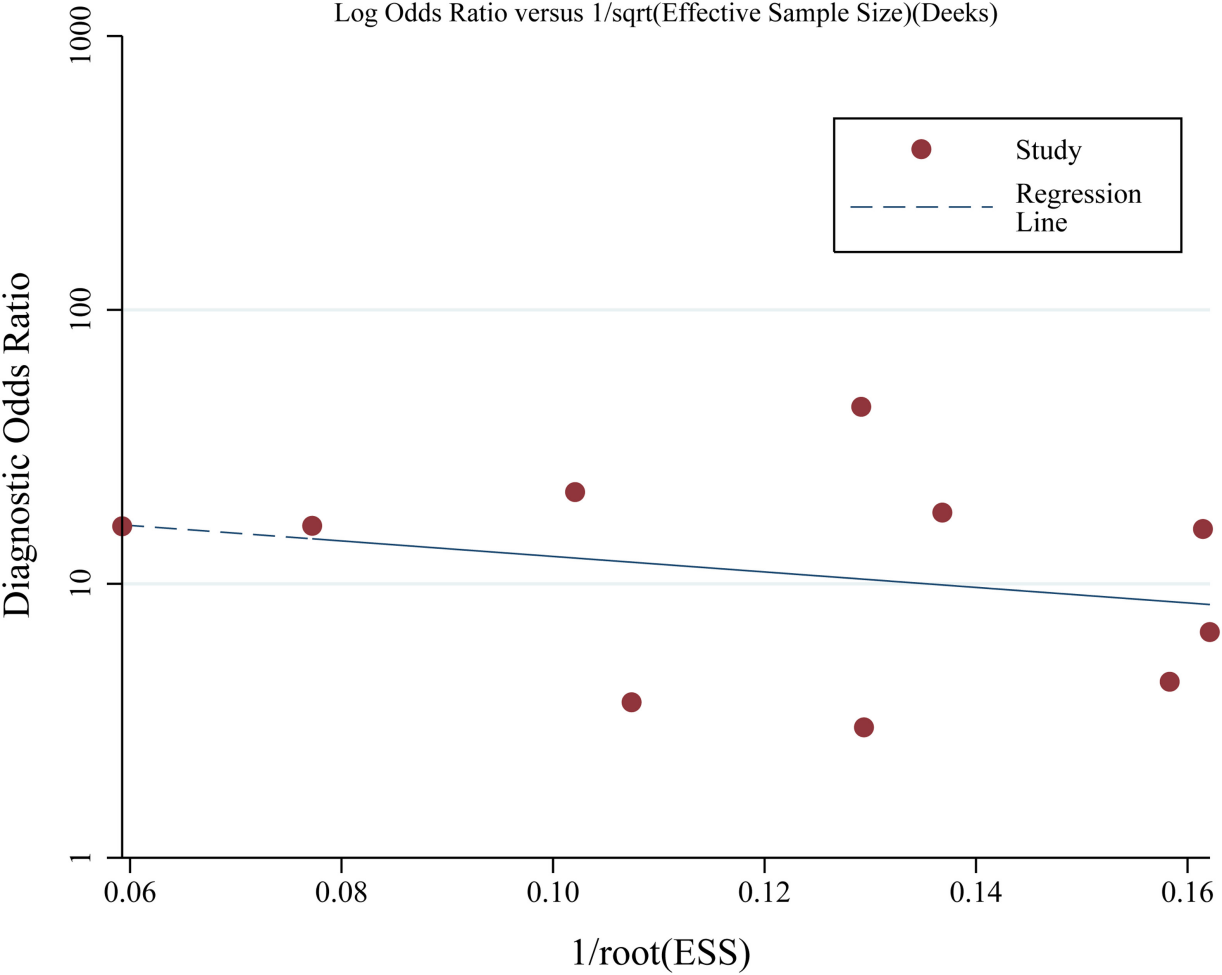
Deeks’ funnel plot assessing potential publication bias in the meta-analysis of elevated blood copeptin levels for predicting mortality in adult patients with sepsis.

The Deeks’ funnel plots for the meta-analyses summarizing the performance of high blood copeptin level for prediction of mortality in adult patients with sepsis are shown in Figure 4, and the asymmetry tests showed a low risk of publication bias (*p* = 0.58).

## Discussion

This meta-analysis demonstrated that elevated blood copeptin levels measured within 48 h of sepsis diagnosis are associated with good prognostic accuracy for predicting short-term mortality in adult patients with sepsis. The pooled sensitivity, specificity, and AUC values indicate that copeptin can identify high-risk patients with reasonable discriminative power, supporting its potential role as an adjunctive biomarker for early risk stratification.

Several biological mechanisms may explain the observed association between elevated copeptin levels and increased mortality in sepsis (14). Copeptin is a stable surrogate marker for AVP release, which is part of the stress response mediated by the hypothalamic–pituitary–adrenal axis (31). In sepsis, systemic inflammation, hypotension, and hypovolemia stimulate AVP secretion, which plays a role in vasoconstriction and water retention (32). Persistently high copeptin levels may reflect sustained activation of neuroendocrine stress pathways, indicating severe physiological derangement (33, 34). Excessive AVP activity can contribute to microcirculatory dysfunction, impaired tissue perfusion, and organ injury, thereby worsening outcomes (35, 36). Moreover, high copeptin levels may parallel elevated inflammatory cytokines, increased oxidative stress, and metabolic disturbances, which are all associated with greater disease severity and mortality risk in septic patients (14, 37). It is also important to recognize that copeptin is not specific to sepsis but reflects a generalized stress response of the hypothalamic–pituitary–adrenal axis. Elevated copeptin levels have been associated with adverse outcomes in several other acute and chronic conditions, including cardiac surgery patients, where baseline copeptin predicted both 30-day and 1-year all-cause mortality (38). These findings suggest that higher copeptin levels may indicate impaired physiological reserve and heightened systemic stress, rather than being unique to sepsis-related pathophysiology. Accordingly, copeptin should be interpreted as a non-specific but clinically relevant marker of illness severity, which may support—but not replace—comprehensive clinical assessment and established prognostic tools.

Considerable heterogeneity, particularly in specificity (I^2^ = 86%), was observed. This likely reflects variability in sepsis diagnostic criteria (Sepsis-1.0 to Sepsis-3.0), assay platforms used to measure copeptin (ELISA, ILMA, FIA), and differences in patient populations and illness severity across study settings. Although we performed a subgroup analysis stratified by cutoff thresholds (≤ 50 vs. > 50 pmol/L), which showed similar diagnostic performance across categories, this did not fully explain the heterogeneity. Subgroup analyses by sepsis definition, assay method, or patient characteristics were not feasible due to the limited number of studies within each subgroup and insufficiently reported clinical details. Moreover, because only study-level dichotomized data were available, copeptin could not be evaluated as a continuous variable. Future individual patient data meta-analyses are needed to determine optimal cutoff values and explore dose–response relationships. Importantly, we observed considerable variation in the copeptin thresholds used across studies, ranging from approximately 11 to 145 pmol/L. While our subgroup analysis showed generally similar accuracy between lower and higher cutoff ranges, the lack of individual patient data precluded identification of a definitive threshold associated with the best predictive performance. This absence of a standardized reference value substantially limits the biomarker’s clinical utility. Future multicenter studies with patient-level analyses are warranted to establish optimal and clinically validated cutoff values to facilitate practical application in sepsis care. On the other hand, although copeptin was consistently measured early, the exact timing (admission to 48 h) varied, and this may have influenced measured concentrations. Standardization of sampling time should therefore be prioritized in future studies.

This study has several strengths. First, it is the most up-to-date quantitative synthesis of the prognostic value of copeptin in adult sepsis. Moreover, the included studies were conducted in multiple countries with different healthcare systems, enhancing the generalizability of the findings. Third, all the included studies were of prospective design, minimizing the risk of recall and selection bias. However, several limitations should be acknowledged. Considerable heterogeneity was observed in specificity estimates, which may be due to differences in sepsis definitions, patient populations, timing of blood sampling, assay methods, and copeptin cutoff values. The small sample sizes in some studies may have led to imprecise estimates and overestimation of effect sizes. Although all studies measured copeptin early after diagnosis, the exact timing varied, which could influence prognostic accuracy due to dynamic changes in biomarker levels. The cutoff thresholds for defining “high” copeptin differed substantially across studies, limiting the ability to recommend a universal reference value for clinical practice. In addition, the included studies did not consistently adjust for potential confounders such as severity scores, comorbidities, or other prognostic biomarkers, which could affect the independent predictive value of copeptin. Finally, all included studies evaluated only short-term mortality (≤ 30 days or in-hospital death), and none reported long-term outcomes. Therefore, the prognostic value of copeptin beyond the acute phase of sepsis remains unknown, and the present conclusions should be interpreted strictly in the context of early mortality risk.

While this meta-analysis provides pooled diagnostic accuracy estimates for copeptin, it does not allow assessment of its clinical utility in decision-making. Decision curve analysis (DCA) quantifies the net clinical benefit of incorporating a biomarker into risk stratification but requires individual patient–level predicted probabilities, which were not available in the included studies as copeptin was reported using study-specific dichotomized thresholds (39). As such, DCA could not be conducted in the present analysis. Future studies and individual participant data meta-analyses should evaluate copeptin within multivariable clinical prediction models and assess its decision-making value using DCA frameworks to determine whether its use improves patient outcomes.

From a clinical perspective, the findings suggest that copeptin could be incorporated into multimodal prognostic assessment in sepsis. Given its ease of measurement, copeptin testing could be implemented alongside clinical scoring systems to enhance early identification of high-risk patients who may benefit from closer monitoring, aggressive hemodynamic support, or early escalation of care (40). This could be particularly valuable in emergency and critical care settings, where rapid and accurate risk stratification is essential for guiding treatment decisions. However, before routine adoption, further research is needed to determine optimal cutoff values, evaluate cost-effectiveness, and assess whether copeptin-guided interventions improve patient outcomes. Future studies should aim to standardize copeptin measurement protocols, including timing of sampling and analytical methods, to reduce heterogeneity and facilitate comparability across studies. Large-scale, multicenter prospective studies are needed to validate the prognostic performance of copeptin across different sepsis phenotypes and healthcare settings. It would also be valuable to investigate whether combining copeptin with other biomarkers, such as procalcitonin, lactate, or mid-regional pro-adrenomedullin, could improve predictive accuracy beyond individual markers (8). Additionally, integrating copeptin into existing sepsis risk scores may enhance prognostic precision and should be tested in clinical trials. Research into the pathophysiological role of copeptin in sepsis could also uncover therapeutic implications, particularly if modulation of AVP-related pathways proves beneficial. Due to the complexity of the pathogenesis of sepsis, copeptin is unlikely to function as a standalone decision-making tool. However, its rapid measurement in blood suggest that it may be incorporated alongside existing severity scores (e.g., SOFA or APACHE II) to improve early risk stratification. In practice, an elevated copeptin level could prompt more frequent vital sign reassessment, earlier hemodynamic optimization, and closer ICU monitoring, particularly in settings where sepsis severity is initially uncertain. Such risk-adapted management may help clinicians allocate resources more efficiently during the early phase of sepsis care. Nevertheless, no current evidence supports altering specific antimicrobial, fluid, or vasopressor strategies solely based on copeptin levels. Thus, copeptin should be considered a complementary marker rather than a directive therapeutic indicator. Future interventional studies are needed to determine whether copeptin-guided escalation of monitoring or supportive therapy improves patient outcomes.

## Conclusion

In conclusion, this meta-analysis provides evidence that elevated blood copeptin levels measured early in the course of sepsis are associated with good prognostic accuracy for short-term mortality in adult patients. These findings highlight copeptin as a promising biomarker for risk stratification, which may complement established clinical tools and facilitate timely, targeted management strategies. Standardization of measurement, determination of optimal thresholds, and prospective validation in diverse patient cohorts are essential next steps to confirm its clinical utility and support its integration into sepsis care pathways.

## Data Availability

The original contributions presented in this study are included in this article/Supplementary material, further inquiries can be directed to the corresponding author.
