# Supplementary material for: Efficacy of blood copeptin level for the prediction of mortality of adult patients with sepsis: a meta-analysis
Source: Front Med (Lausanne). 2026 Feb 6;12:1686137. doi: 10.3389/fmed.2025.1686137 (PMC12920574; doi:10.3389/fmed.2025.1686137)
Supplement: Supplementary file 1 [file Table_1.docx]

**Detailed search strategy for each database**

**PubMed**

("copeptin"[MeSH Terms] OR copeptin[Title/Abstract] OR "C-terminal provasopressin"[Title/Abstract]) AND ("sepsis"[MeSH Terms] OR sepsis[Title/Abstract] OR septic[Title/Abstract] OR septicemia[Title/Abstract])

**Embase**

('copeptin'/exp OR copeptin:ti,ab OR 'c-terminal provasopressin':ti,ab) AND ('sepsis'/exp OR sepsis:ti,ab OR septic:ti,ab OR septicemia:ti,ab)

**Web of Science**

TS = (copeptin OR "C-terminal provasopressin") AND TS = (sepsis OR septic OR septicemia)

**Wanfang Data**

主题: ("copeptin" OR "C末端加压素原肽" OR "和肽素") AND 主题: ("脓毒症" OR "脓毒" OR "败血症")

**CNKI (China National Knowledge Infrastructure)**

主题: ("copeptin" OR "C末端加压素原肽" OR "和肽素") AND 主题: ("脓毒症" OR "脓毒" OR "败血症")
